# Upf2-Mediated Nonsense-Mediated Degradation Pathway Involved in Genetic Compensation of *TrpA1* Knockout Mutant Silkworm (*Bombyx mori*)

**DOI:** 10.3390/insects15050313

**Published:** 2024-04-26

**Authors:** Dong-Yue Wang, Juan Zhu, Yi-Zhong Zhang, Qian-Yi Cui, Shan-Shan Wang, Yang-Wei Ning, Xing-Jia Shen

**Affiliations:** 1 Jiangsu Key Laboratory of Sericultural and Animal Biotechnology, School of Biotechnology, Jiangsu University of Science and Technology, Zhenjiang 212100, China; wdy15225644315@163.com (D.-Y.W.); zhangyizhongccc@163.com (Y.-Z.Z.); cuiqy0103@163.com (Q.-Y.C.); wss10032022@163.com (S.-S.W.); n19709641831@163.com (Y.-W.N.); 2 Key Laboratory of Silkworm and Mulberry Genetic Improvement, Ministry of Agriculture and Rural Affairs, Sericultural Scientific Research Center, Chinese Academy of Agricultural Sciences, Zhenjiang 212100, China

**Keywords:** *BmTrpA1*, CRISPR/Cas9, *Bombyx mori*, genetic compensation response, nonsense-mediated mRNA degradation pathway

## Abstract

**Simple Summary:**

This study, focusing on silkworms as the research subject, investigated the detrimental effects of gene mutants containing premature termination codons on organisms and the potential genetic compensation effects they may trigger. The research findings revealed the presence of genetic compensation effects in silkworms with a premature termination mutation in the *BmTrpA1* gene, leading to a significant increase in the mRNA levels of BmTrpA subfamily paralogs and in Upf2, a key factor of the nonsense-mediated mRNA degradation (NMD) pathway. Knocking down the key factor could suppress the genetic compensation effects. These discoveries document genetic compensation and its role in Lepidoptera insects.

**Abstract:**

Genetic mutations leading to premature termination codons are known to have detrimental effects. Using the Lepidoptera model insect, the silkworm (*Bombyx mori*), we explored the genetic compensatory response triggered by mutations with premature termination codons. Additionally, we delved into the molecular mechanisms associated with the nonsense-mediated mRNA degradation pathway. CRISPR/Cas9 technology was utilized to generate a homozygous bivoltine silkworm line BmTrpA1^−/−^ with a premature termination. Transcript levels were assessed for the *BmTrpA* paralogs, *BmPyrexia* and *BmPainless* as well as for the essential factors Upf1, Upf2, and Upf3a involved in the nonsense-mediated mRNA degradation (NMD) pathway. Upf2 was specifically knocked down via RNA interference at the embryonic stage. The results comfirmed that the *BmTrpA1* transcripts with a 2-base deletion generating a premature termination codon in the BmTrpA1^−/−^ line. From day 6 of embryonic development, the mRNA levels of *BmPyrexia*, *BmPainless*, *Upf1*, and *Upf2* were significantly elevated in the gene-edited line. Embryonic knockdown of Upf2 resulted in the suppression of the genetic compensation response in the mutant. As a result, the offspring silkworm eggs were able to hatch normally after 10 days of incubation, displaying a non-diapause phenotype. It was observed that a genetic compensation response does exist in BmTrpA1^−/−^ *B. mori*. This study presents a novel discovery of the NMD-mediated genetic compensation response in *B. mori*. The findings offer new insights into understanding the genetic compensation response and exploring the gene functions in lepidopteran insects, such as silkworms.

## 1. Introduction

The silkworm, *Bombyx mori*, is an important agricultural and economic insect, and the identification of its genes related to its economic traits holds paramount significance for sericulture production and even agricultural economy development. However, with the increasing maturity and widespread application of reverse genetics technology, such as TALENs and CRISPR, the genetic compensation response (GCR) has been hindering the study of gene function [1]. The GCR refers to the function mutant that is gained by knockout of the target gene; the deleterious mutation can lead to the transcriptional upregulation of compensatory genes, which ultimately results in a null mutant with a relatively normal phenotype. The GCR phenomenon has been observed in various model organisms, such as Arabidopsis [2], mice [3], drosophila [4], zebrafish [5], and human cell lines [6], revealing the molecular mechanisms of genetic compensation effects. Through the use of genetic mutants with variations in different genes as models, it has been substantiated that the activation of genetic compensation effects relies on two key prerequisites: the occurrence of nonsense mutations and sequence homology similarity to the knocked-out gene. The nonsense-mediated mRNA degradation pathway (NMD) is involved in triggering genetic compensation effects [7,8]. Furthermore, it has been observed that the high expression of compensatory genes is associated with the H3K4me3 modification at their transcription start sites [9]. However, there are different views on whether the GCR is triggered by the Upf1 or Upf3a degradation factor in the NMD degradation pathway. It is proposed that the degradation factor Upf3a in NMD is involved in homology-dependent genetic compensation effects [10,11], while it is also suggested that the key NMD degradation factor Upf1 participates in genetic compensation effects [9].

The transient receptor potential A1 (TRPA1) channel, expressed at the terminals of sensory nerves, is a non-selective cation channel with good permeability to Ca^2+^, widely distributed in both neural and non-neural cells. Its function is regulated by a variety of factors, acting as a detector for temperature, chemical, and mechanical stimuli [12,13]. In insects, the TRPA subfamily serves as a temperature-sensitive channel, receiving environmental signals to further regulate physiological activities in individuals [14,15,16,17,18]. RNA interference on the *TRPA1* gene of the silkworm, *B. mori* (*BmTrpA1*), in the embryonic stage, followed by incubation in the dark at 25 °C, resulted in the partial production of non-diapause eggs in the offspring [19]. Further confirmation through techniques such as patch-clamp and calcium imaging revealed that TRPA1, as a temperature-sensitive channel, was activated at temperatures above 21 °C during the embryonic stage and that the signaling pathway associated with diapause hormone (DH) release was also activated. Additionally, knockout of the *BmTrpA1* gene extended the larval growth period of the silkworms at a temperature of 29 °C, suggesting the involvement of BmTrpA1 in regulating larval growth and development [20]. However, the mechanism by which the *BmTrpA1* gene regulates the development of the larva of silkworm remains unclear.

To elucidate the key signaling pathways through which the TRPA1 protein regulates diapause in silkworms, B. mori, we first constructed a TrpA1 mutant, i.e., BmTrpA1^−/−^, with the CRISPR/Cas9 technique. However, unlike the results after RNAi of *BmTrpA1*, the eggs of BmTrpA1^−/−^ did not exhibit the phenotype of non-diapause eggs. The mutant carried a premature termination codon (PTC) in TrpA1, consistent with the degradation of nonsense-mediated mRNA. Therefore, we speculate that there are genetic compensation effects in the mutant. Consequently, we conducted research related to genetic compensation. The real-time fluorescence quantification results revealed an upregulation in the expression levels of the *BmTrpA* subfamily gene and the related factors involved in the nonsense degradation pathway during the embryonic stage of the mutant. Finally, we performed RNAi experiments in the *Upf2* gene, and the results indicate that the Upf2 factor plays a crucial role in the genetic compensation pathway of the silkworm. This study represents the first discovery of the genetic compensation effects in the *lepidopteran* insect *B. mori*. This finding contributes to elucidating the genetic compensation pathways of *B. mori* and provides a theoretical foundation and new perspectives for establishing functional gene identification systems for *B. mori* and other insects.

## 2. Materials and Methods

### 2.1. Silkworm, B. mori

The bivoltine strain Qiufeng of *B. mori*, characterized by sex-limited markings on its larval body, was utilized in this study and has been meticulously maintained by the Key Laboratory of Silkworm and Mulberry Genetic Improvement, Ministry of Agriculture. Diapause-terminated eggs of Qiufeng were incubated at a temperature of 16 °C to 17 °C under continuous darkness throughout embryogenesis to yield non-diapause eggs in the next generation, suitable for gene editing via microinjection. Subsequently, the larvae hatched from the genetically edited eggs were raised on fresh mulberry leaves at 25 °C under a 12 h light/dark cycle with a relative humidity of 70% to 80% [21]. For other experiments, the silkworm eggs were incubated at 25 °C with a relative humidity of 70% to 80% under darkness.

### 2.2. Construction of the BmTrpA1^−/−^ Mutant and Prediction of the Three-Dimensional Structure of the Protein

The CDS sequence and genomic sequence of *BmTrpA1* (LOC101744290) were downloaded from the NCBI website, and sgRNA target sites were designed using the online software CRISPRdirect (http://crispr.dbcls.jp/, accessed on 19 April 2021) (shown in Table 1). According to the instructions of the EnGen^®^ sgRNA Synthesis Kit for *S. pyogenes* (NEB #E3322), the sgRNA was synthesized and mixed with the Cas9 protein EnGen^®^ Spy Cas9 NLS (NEB #M0646) at a molar ratio of 1:1 and used for microinjection of the non-diapause eggs after incubation at 37 °C for 5 min [22]. After injection, the silkworm eggs were transferred to an incubator with the temperature set to 25 °C, in darkness, and with a relative humidity of 80%. After hatching, the larvae were fed fresh mulberry leaves at 25 °C ± 1 °C.

Snap Gene^®^3.2.1 software was used to derive the corresponding amino acid sequence and alignment. The three-dimensional structure of the protein was obtained by the homology modeling method and predicted by the online software Swiss-Model (http://swissmodel.expasy.org/, accessed on 11 July 2023) [23,24].

### 2.3. Mutant Screening

The microinjected eggs (G0 generation) were hatched at 25 °C and reared on fresh mulberry leaves. Then, the male and female moths of G0 were numbered and mated with each other. The genomic DNA of each moth was extracted, and PrimerSTAR^®^ HS DNA polymerase (Takara, Shiga, Japan) was used to amplify the 500 bp fragment around the target, with the PCR primers shown in Table 1. The knockout chimeras were screened by genome sequencing, followed by continuous sibling mating and sequencing selection until obtaining an egg batch of which both parents were homozygous mutants. Subsequently, sibling mating breeding was continued to obtain the mutant homozygous strain, i.e., BmTrpA1^−/−^.

### 2.4. Investigation of Larval Growth and Development and Cocoon Economic Traits

Totals of 30 wild-type (wt) larvae and 30 mutant larvae were reared in each group, with 3 replicates. Their growth and development were investigated. The growth status of each individual in the population was observed every 12 h from the molting of the third instar larvae. When the larvae transitioned from one instar to the next, the proportion of larvae entering the next instar was investigated [25]. After cocoon harvesting, the cocoon economic traits were investigated.

### 2.5. Extraction of Total RNA from Silkworm Tissues and RT-qPCR Analysis

The total RNA from the tissues of the silkworms was extracted using RNAiso Plus (Takara, Shiga, Japan) according to the given protocol and stored at −80 °C until use. The RNA was quantified by measuring its absorbance at 260 nm using a Nanodrop 1000 spectrophotometer (GE Healthcare, Chicago, IL, USA), and the purity of the RNA sample was assessed based on the absorbance ratio of OD260/280 and OD260/230. The RNA was further confirmed to be intact via electrophoresis on 1% agarose gels. The HI Script 1st Strand cDNA Synthesis Kit (Vazyme, Nanjing, China) was used to synthesize the complementary DNA according to the manufacturer’s instructions. To design the primers (Table 2), we used Oligo 7.0 software. We performed quantitative real-time PCR assays using TB Green™ Premix ExTaq™ II (Takara, Shiga, Japan) with the Quant Studio™ 6 Flex Real-Time PCR System (Applied Biosystems, Carlsbad, CA, USA) and confirmed that the amplicon efficiencies were approximately equal and between the values of 0.8 and 1. The 2^−ΔΔCt^ method [26] was employed to calculate the relative gene expression levels, and the internal reference genes used were *Rp49* and *Actin3*.

We conducted three biological replicates of each reaction, each of which was subjected to three technical replicates. The significance of the differences was analyzed using GraphPad Prism 8.0 software and evaluated using *t*-tests. A probability value of less than 0.05 was considered statistically significant, while values of less than 0.01 were considered extremely significant.

### 2.6. Embryonic RNA Interference

RNAi procedures were adapted from Masumoto et al. [27], with some modifications. The synthesis of dsRNA was performed by the T7 RiboMAX Express RNAi System (Promega) using the primers shown in Table 3. The concentration of dsRNAs was adjusted to 2.5 μg/μL. The BmTrpA1^−/−^ eggs were incubated at 16 °C to 17 °C under continuous darkness throughout embryogenesis to obtain non-diapause eggs in the subsequent generation. For injection into the eggs, non-diapause eggs of the BmTrpA1^−/−^ were collected within 2 h of oviposition during the syncytial blastoderm stage. Then, dsRNA was injected into the eggs using a glass needle attached to a capillary puller (PC-100; NARISHIGE, Solana Beach, CA, USA) and FemtoJet (Eppendorf, Oldenburg, Germany). The eggs injected with dsRNA were incubated at 25 °C in darkness with a relative humidity of 80%, and after hatching, the larvae were fed fresh mulberry leaves at 25 °C ± 1 °C. The control group was replaced with an equal amount of dsGFP [28].

## 3. Results

### 3.1. Construction of a BmTrpA1^−/−^ Mutant Strain

**Figure 1:**
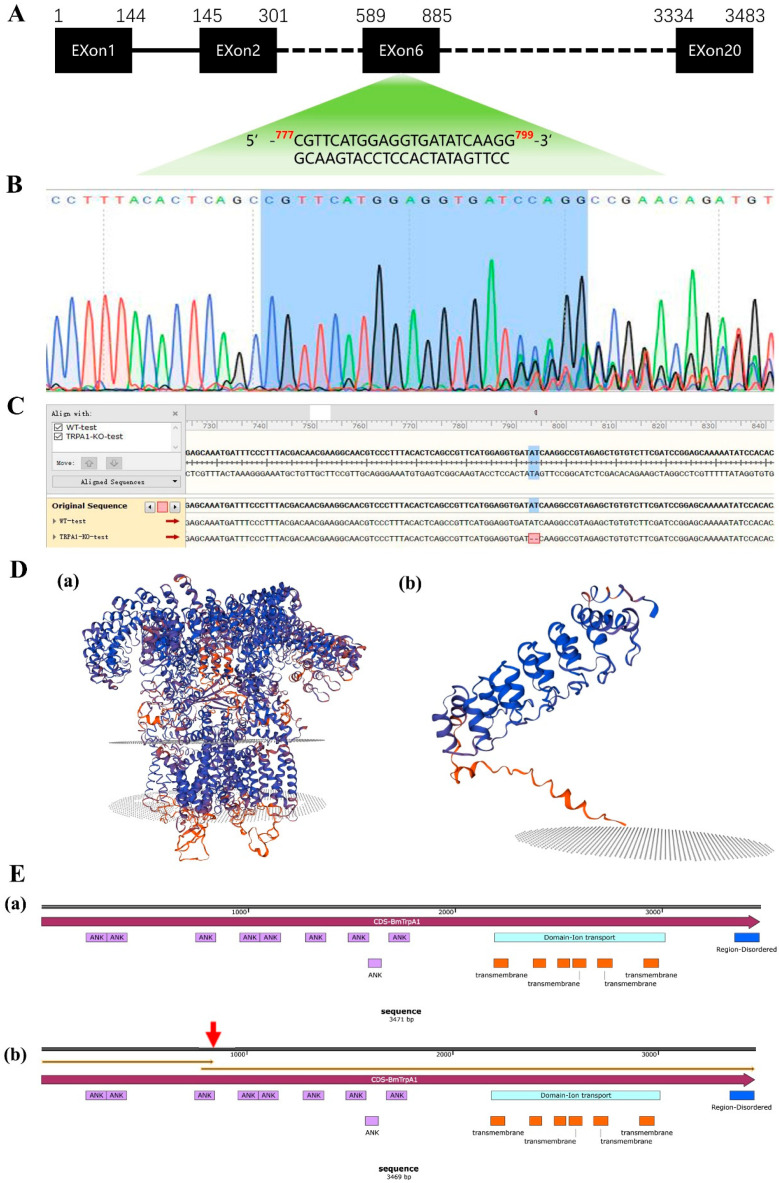
Screening of *BmTrpA1* gene knockout target sites and mutants. (**A**) *BmTrpA1* gene editing target site; (**B**) sequencing peak chart of G0 generation positive individual; (**C**) sequence alignment chart of the G5 generation mutation homozygous and wild-type silkworm; (**D**) protein three-dimensional structure prediction ((**a**) wild-type, (**b**) mutant); (**E**) *BmTrpA1* sequence information and protein characteristic information. (**a**) *BmTrpA1* gene CDS sequence and protein information, (**b**) CDS sequence and protein information of *BmTrpA1* gene after deleting two bases. The place marked by the red arrow is the position where the translation of the *BmTrpA1* gene terminates prematurely. The orange box represents the transmembrane region. The red arrow indicates the premature termination codon.

The target sites were designed using the online software CRISPRdirect (http://crispr.dbcls.jp/ accessed on 24 April 2024), and they were located in the sixth exon (Figure 1A) with the sequence CGTTCATGGAGGTGATATCAAGG. One positive individual was obtained in the G0 mass through sequencing screening, with overlapping peaks appearing at the NGG site in the sequencing chromatogram (Figure 1B). Sibling mating and sequencing screening were conducted continuously from the G_1_, and ultimately, the *BmTrpA1* knockout homozygote BmTrpA1^−/−^ was obtained in the G_5_ mass. This homozygous mutant had a deletion of two bases at +787 bp and +788 bp (Figure 1C), resulting in a premature stop codon at +843 bp in the BmTrpA1^−/−^. The three-dimensional structure of wt and mutant TRPA1 proteins were predicted with the online SWISS-MODEL software. There is an obvious change in the three-dimensional structure of the mutant TRPA1 protein compared to that of the wt (see Figure 1D). Further analysis of the encoded protein sequences and features showed that neither the repetitive anchoring protein sequence nor the functional domain responsible for the Ca^2+^ transport channel lost their function in the mutant BmTrpA1 (see Figure 1E).

### 3.2. No Obvious Differences Were Observed in the Growth, Development, or Diapause Phenotype between BmTrpA1^−/−^ and wt

**Figure 2:**
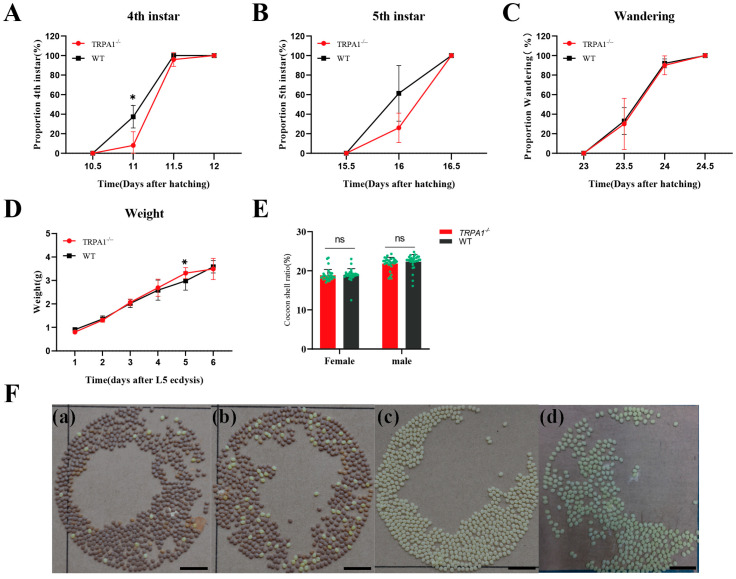
Analysis of growth and development traits, economic traits, and diapause phenotypes of mutant and wild-type silkworms. (**A**–**C**) Ratio of *BmTrpA1* mutant silkworms to wild-type silkworms molting into the 4th instar (**A**), 5th instar (**B**), and wandering stage (**C**). (**D**) Daily body weight changes in *BmTrpA1* mutant silkworm and wild-type silkworm after the fourth molt. Each group had 10 biological replicates, and error bars are drawn with standard errors. (**E**) The cocoon shell rate of mutant silkworms and wild-type silkworms; 30 biological replicates were taken for each group, and the error bars are drawn with standard errors. (**F**) Comparison of diapause eggs and non-diapause eggs. (**a**) Color of eggs laid by wild-type silkworms incubated at 25 °C after 48 h. (**b**) Color of eggs laid by mutant silkworms incubated at 25 °C after 48 h. (**c**) Color of eggs laid by mutant silkworms incubated at 15 °C after 48 h. (**d**) Color of eggs laid by wild-type silkworms incubated at 15 °C after 48 h. The significance of the differences was analyzed by a two-tailed *t*-test. * *p* < 0.05. Scale bars represent 10 mm.

In this study, we compared the larvae of BmTrpA1^−/−^ and the wild-type, and there was no significant difference in the developmental duration between the 4th and 5th instar, nor in the wandering stage (Figure 2A–C). Furthermore, in the 5th instar, there was also no significant difference in the body weight between the BmTrpA1^−/−^ larvae and the wt larvae (Figure 2D). A significant difference was detected in the cocoon weight between the mutant males and the wt males, while no difference was found in the cocoon shell weight. Similarly, when comparing the mutant female pupae with the wt females, no significant differences were detected in either the cocoon weight or cocoon shell weight (Table 4). The cocoon shell ratio showed no significant difference between the mutants and the wt (Figure 2E).

The diapause-terminated eggs of the mutants and wt were incubated at 25 °C under darkness. After hatching, the larvae were normally fed fresh mulberry leaves. After eclosion, female moths were mated with males of the same strain. Both the wild-type and mutant female moths produced diapause-destined eggs, which turned a light purple color at 48 h post-oviposition and eventually displayed the characteristic purple-brown color of diapause eggs (Figure 2F(a,b)). In contrast, moths developed from the embryos incubated under darkness at 15 °C produced non-diapause eggs of both the mutants and wt. The color of these eggs changed from light yellow to green and brown over a span of 1 to 7 days, eventually hatching on the 10th day (Figure 2F(c)). No significant differences were observed in the diapause characteristics between the wt and mutants.

### 3.3. BmPyrexia, BmPainless, and BmUpf2 Showed Upregulated Expression in BmTrpA1^−/−^ Mutant

**Figure 3:**
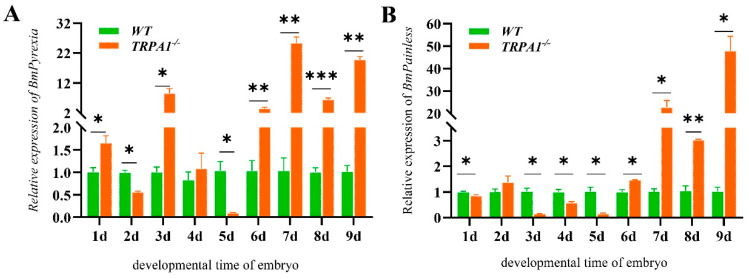
The relative expression of mRNA of the *BmTRPA* subfamily genes at the silkworm embryonic stage. (**A**) The relative expression of *BmPyrexia* at the embryonic stage, days 1–9. (**B**) The relative expression of *BmPainless* at the embryonic stage, days 1–9. Each entry represents the mean ± standard deviation of three samples. * *p* < 0.05; ** *p* < 0.01; *** *p* < 0.001.

**Figure 4:**
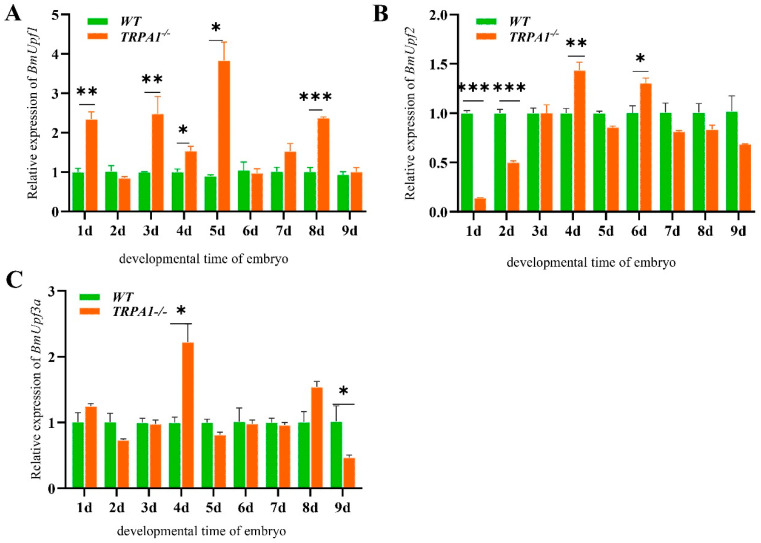
The relative expression levels of *BmUpf1*, Bm*Upf2*, and Bm*Upf3a* at the silkworm embryonic stage. (**A**) The relative expression of *BmUpf1* at the embryonic stage, days 1–9. (**B**) The relative expression of *BmUpf2* at the embryonic stage, days 1–9. (**C**) The relative expression of *BmUpf3a* at the embryonic stage, days 1–9. Each entry represents the mean ± standard deviation of the expressions of three samples. * *p* < 0.05; ** *p* < 0.01; *** *p* < 0.001.

The expression levels of *BmPyrexia*, *BmPainless*, *BmUpf1*, *BmUpf2*, and *BmUpf3a* of the eggs were analyzed by RT-qPCR from the 1st to 9th day post-oviposition. The results show that the expression level of *BmPyrexia* was significantly higher in the eggs of the mutants than those of the wt on day 1 and day 3, and increased significantly from day 6 to day 9 in the eggs of the mutants (Figure 3A). *BmPainless* showed significantly lower expression levels in the eggs of the mutants than those of the wt on day 1 and days 3 to 5, and they increased significantly from day 6 to day 9 in the mutants. Compared to the wt, the expression levels of *BmPainless* in the eggs of the mutants on day 8 were significantly upregulated (Figure 3B). The mRNA expression levels of *BmUpf1* on day 1, days 3 to 5, and day 8 were also significantly upregulated in the eggs of the mutants (Figure 4A). The *BmUpf2* expression levels on the first and second day were significantly lower in the eggs of the mutants than those of the wt, while on day 4 and day 6, the expression levels of *BmUpf2* were significantly higher in the eggs of the mutants than those of the wt (Figure 4B). Meanwhile, the relative expression level of *BmUpf3a* in the mutants showed no significant difference from that of the wt for most of the time, except for a significant increase on day 4 (Figure 4C).

### 3.4. RNA Interference of the UPF2 Gene Alters the Mutant’s Diapause Phenotype

**Figure 5:**
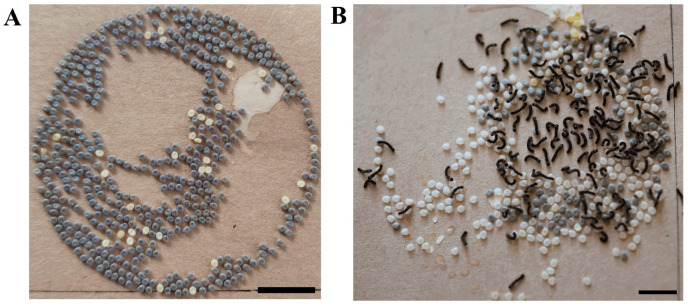
Effects of injection of dsUpf2 in diapause eggs of mutant silkworms. (**A**) The 10th day after oviposition in the dsGFP control group. (**B**) The 10th day after oviposition in the dsUpf2 group. Scale bars represent 10 mm.

The non-diapause freshly eggs laid by the mutants were microinjected with synthesized dsUpf1 and dsUpf2 separately, while the eggs of the control group were microinjected with dsGFP, and the injected eggs were incubated at 25 °C under darkness then reared to adulthood for mating. The eggs laid after mating are placed in a 25 °C incubator. On the 10th day, compared to the control group, the eggs produced by the moth microinjected with dsUpf2 in the incubation started hatching (shown in Figure 5), while the eggs produced by moths microinjected with dsUpf1 entered the diapause period. The results indicate that the eggs of the mutants injected with dsUpf2 in the early embryo stage changed from diapause-destined eggs to partially non-diapause eggs.

## 4. Discussion

Hundreds of mutant strains of the silkworm *B. mori*, as a model organism of *Lepidoptera*, have emerged as excellent resources for modern genetic engineering research. With the completion of the silkworm pan-genome and multi-omics data assembly, the silkworm is increasingly being recognized as an ideal model organism for investigating functional genomics, genome selection, and molecular breeding, among other aspects, in the field of insect research. The study successfully generated a knockout mutant of the *BmTrpA1* gene using CRISPR/Cas9 technology, resulting in a premature stop codon at the 281st codon of the *BmTrpA1* transcript, i.e., a nonsense mutation. By analyzing the subfamily genes of BmTrpA, it was found that the expressions of two genes, *BmPyrexia* and *BmPainless*, were upregulated during the temperature-sensitive period, suggesting that the lack of change in the diapause phenotype resulted from these two BmTrpA subfamily genes compensating for the loss of BmTrpA1 function. Furthermore, by downregulating the degradation factors of NMD in the mutants, it was observed that the mutants with lower Upf2 expression exhibited a non-diapause phenotype. This indicates that the NMD degradation pathway mediated by Upf2 is involved in genetic compensation effects in the silkworm.

The synthesis and release of diapause hormone (DH) in silkworms are regulated by a network system composed of the γ-aminobutyric acid (GABA)ergic and the corazonin signaling pathway [29]. The activation of BmTrpA1 at a 25 °C induction temperature led to the transcriptional expression of the silkworm *GAT* gene through an unknown pathway, inhibiting the GABAergic signal, which accelerated the release of corazonin in the dorsal lateral neurons, thereby inducing the release of DH in the silkworm and resulting in progeny diapause. In addition, 3-hydroxykynurenine is the primary component responsible for the pigmentation of diapause eggs, whereas this pigment is absent in non-diapause eggs. We successfully deleted two bases, 793 bp and 794 bp, on the 6th exon of the *BmTrpA1* gene. After obtaining positive individuals in the G0 mass, we established the BmTrpA1^−/−^ homozygous mutant in the 5th generation through sibling mating and test crossing. Eggs laid by the homozygous mutant moths under the diapause induction temperature (25 °C) were observed and compared with the diapause and non-diapause eggs of the wt. It was found that the eggs still exhibited a purplish-brown color, indicative of diapause eggs. The diapause phenotype of the mutant silkworms did not transition to non-diapause, which is inconsistent with the experimental results of *BmTrpA1* knockdown leading to non-diapause [19]. It is speculated that other genes may have compensated for the loss of BmTrpA1 function. Furthermore, the transcription levels of the BmTrpA subfamily genes *BmPyrexia* and *BmPainless* during the embryonic stage revealed a significant increase during the temperature-sensitive period (day 6 to day 7) in the mutant embryo. Additionally, the expressions of the degradation factors Upf1 and Upf2 in the NMD pathway were also significantly upregulated in the mutants. It is, therefore, conjectured that in the *BmTrpA1* knockout mutants, the upregulation of the subfamily genes *BmPyrexia* and *BmPainless* compensated for the loss of BmTrpA1 function, resulting in no change in the diapause phenotype of the mutants. Further confirmation was provided by knocking down *BmUpf1* and *BmUpf2* in the BmTrpA1^−/−^, demonstrating that the genetic compensatory effect in silkworm is mediated by Upf2, rather than Upf1 or Upf3a. It is speculated that the function of Upf2 in the NMD degradation pathway in silkworm differs from the organisms of other species. Upf2 is a core scaffold protein in the NMD surveillance pathway, with its N-terminus containing three middle domains of the eukaryotic initiation factor 4G (mIF4G-1, mIF4G-2, and mIF4G-3), playing an important role in regulating the surveillance pathway [30,31]. In particular, the intrinsically charged residues within the mIF4G-1 of Upf2p play a regulatory role in the NMD surveillance mechanism in budding yeast. Early studies have found that in human cell lines and zebrafish, knockout bodies with premature termination codons led to the upregulation of subfamily gene expression after gene knockout, suggesting the existence of a genetic compensation effect in which the NMD degradation pathway is involved. In human cells, the activation of GCR requires the Upf3a-mediated NMD degradation pathway, rather than the Upf1-mediated NMD degradation pathway. However, in zebrafish, it is believed to be mediated by Upf1 rather than Upf3a [10].

In this study, we knocked down the key factors of NMD in the BmTrpA1^−/−^ mutant, resulting in the blockade of genetic compensation effects. However, further knockdown of other key factors in the NMD pathway is needed to validate their specific roles in genetic compensation effects. In the present study, a mutant containing a premature termination codon has been successfully established in silkworm, and preliminary evidence has indicated the involvement of the Upf2-mediated NMD degradation pathway in the genetic compensation effects. More unknown facts are still needed to explain how the NMD degradation pathway regulates the upregulation of subfamily genes after gene knockout, and the molecular mechanism between them is currently unclear. Our study has indicated that Upf2 plays a key role in activating genetic compensation effects, rather than Upf1 or Upf3a; however, there are still some details and in-depth mechanisms that need further refinement. By blocking the genetic compensation effects, it is possible to more reliably reveal the potential new functions of genes, which can help advance the research on gene function.

## 5. Conclusions

This study successfully generated a mutant silkworm with a premature termination codon in the *BmTrpA1* gene using CRISPR/Cas9 technology. It is tentatively believed that genetic compensation effects exist in the lepidopteran model insect, silkworm *B. mori*. *BmTrpA1* knockout mutants have shown genetic compensation effects, in which the Upf2-mediated NMD degradation pathway is involved. These research findings provide us with a new perspective on understanding the genetic compensation effects and gene function research on silkworms and other lepidopteran insects.

## Figures and Tables

**Table 1:** PCR primer sequences.

Primer Name	Primer Sequence (5′–3′)	Primer Purpose
TrpA1-582-F	GATAACCGATCTGGACGCTGAATG	Mutation site sequencing detection
TrpA1-880-R	CTTGAGCGCAAGCTAAGTGCACAG	
gRNA-771-793	TtctaatacgactcactatagCGTTCATGGAGGTGATA TCAgttttagagctaga	Synthesis of sgRNA in vitro

**Table 2:** RT-qPCR primers.

Primer Name	Primer Sequence (5′–3′)	Gene Accession No.
BmPainless-F	CGGTCTTGCGGTTAGTGACA	NM_001309624.1
BmPainless-R	GCTACCGATAAGCACGCTCT	
BmPyrexia-F	ATGATGGCCGCTTACGACAT	NM_001309607.1
BmPyrexia-R	TCCGAGTCCTGAGTAACCGT	
BmUpf1-F	GCGAGAGGCAATGGAGTCTT	XM_004929604.4
BmUpf1-R	CACCGGCTTCTTCCTTGAGT	
BmUpf2-F	CATTGCTGTCCCGATGACCT	XM_038010585.1
BmUpf2-R	AACGAATTCCACGCCCTCTT	
BmUpf3a-F	GGATCGGAAGAGACAGACACA	NM_001046855.2
BmUpf3a-R	TTCTTTCGCGAGACGCTGTT	
BmActin3-F	CGGCTACTCGTTCACTACC	NM_001126254.1
BmActin3-R	CCGTCGGGAAGTTCGTAAG	
BmRp49-F	TCAATCGGATCGCTATGACA	NM_001098282.2
BmRp49-R	ATGACGGGTCTTCTTGTTGG	

**Table 3:** Primers for dsRNA synthesis.

Primer Name	Primer Sequence (5′–3′)
dsGFP-F	ACGTAAACGGCCACAAGTTC
T7dsGFP-F	TAATACGACTCACTATAGGGACGTAAACGGCCACAAGTTC
dsGFP-R	TGTTCTGCTGGTAGTGGTCG
T7dsGFP-R	TAATACGACTCACTATAGGGTGTTCTGCTGGTAGTGGTCG
dsUpf1-F	CTCGCAATCGCTCACGTTTC
T7dsUpf1-F	TAATACGACTCACTATAGGGCTCGCAATCGCTCACGTTTC
dsUpf1-R	ACATTCCGAGCACCACATGA
T7dsUpf1-R	TAATACGACTCACTATAGGGACATTCCGAGCACCACATGA
DsUpf2-F	TCATCAAAACTGCGGGTGGAT
T7dsUpf2-F	TAATACGACTCACTATAGGGTCATCAAAACTGCGGGTGGAT
DsUpf2-R	TGAGAGTTCTCCTCTGGTGTG
T7dsUpf2-R	TAATACGACTCACTATAGGGTGAGAGTTCTCCTCTGGTGTG

**Table 4:** Statistical analysis of silkworm cocoons and pupae quality.

Group	Whole Cocoon Weight of Females (g)	Whole Cocoon Weight of Males (g)	Cocoon Shell Weight of Females (g)	Cocoon Shell Weight of Males (g)
WT (*n* = 30)	1.673 ± 0.1385	1.300 ± 0.06543	0.3189 ± 0.03326	0.2973 ± 0.03197
TRPA1^−/−^(*n* = 30)	1.744 ± 0.1559	1.479 ± 0.1320	0.3014 ± 0.02375	0.299 ± 0.02042
*p* Value	0.0683	<0.0001	0.0225	0.8145

## Data Availability

Most of the analytical data are provided in the article. More original datasets used and/or analyzed during the current study are available from the corresponding author upon reasonable request.
